# Comprehensive characterization of small noncoding RNA profiles in hypoxia-induced pulmonary hypertension (HPH) rat tissues

**DOI:** 10.1016/j.isci.2024.108815

**Published:** 2024-01-13

**Authors:** Jun Wang, Jiahao Kuang, Shasha Zhang, Zixin Liu, Qianwen Guo, Shujin Li, Lin Qiu, Gaohui Fu, Xinyang Lin, Jiayu Wu, Jinglin Tian, Jinyong Huang, Yanqin Niu, Kang Kang, Yunhui Zhang, Deming Gou

**Affiliations:** 1 Shenzhen Key Laboratory of Microbial Genetic Engineering, Vascular Disease Research Center, College of Life Sciences and Oceanography, Guangdong Provincial Key Laboratory of Regional Immunity and Disease, Carson International Cancer Center, School of Medicine, Shenzhen University, Shenzhen 518060, China; 2 College of Medicine, Shenzhen University, Shenzhen 518060, China; 3 College of Physics and Optoelectronic Engineering, Shenzhen University, Shenzhen 518060, China; 4 Department of Pulmonary and Critical Care Medicine, The First People’s Hospital of Yunnan Province, The Affiliated Hospital of Kunming University of Science and Technology, Kunming 650022, China

**Keywords:** Cardiovascular medicine, Transcriptomics, Model organism

## Abstract

Hypoxia-induced pulmonary hypertension (HPH) is a fatal cardiovascular disease characterized by an elevation in pulmonary artery pressure, resulting in right ventricular dysfunction and eventual heart failure. Exploring the pathogenesis of HPH is crucial, and small noncoding RNAs (sncRNAs) are gaining recognition as potential regulators of cellular responses to hypoxia. In this study, we conducted a comprehensive analysis of sncRNA profiles in eight tissues of male HPH rats using high-throughput sequencing. Our study unveiled several sncRNAs, with the brain, kidney, and spleen exhibiting the highest abundance of microRNA (miRNA), tRNA-derived small RNA (tDR), and small nucleolar RNA (snoRNA), respectively. Moreover, we identified numerous tissue-specific and hypoxia-responsive sncRNAs, particularly miRNAs and tDRs. Interestingly, we observed arm switching in miRNAs under hypoxic conditions and a significant increase in the abundance of 5′ tRNA-halves among the total tDRs during hypoxia. Overall, our study provides a comprehensive characterization of the sncRNA profiles in HPH rats.

## Highlights

• Tissue-specific and hypoxia-responsive sncRNAs were identified in HPH rats
• Brain, kidney, and spleen had highest abundance of miRNA, tDR, and snoRNA
• Arm switch of miRNAs and increased 5′ tRNA halves were observed under hypoxia
• CeRNA network showed miRNAs regulate extracellular matrix genes in pulmonary artery remodeling

## Graphical abstract

**Figure:**
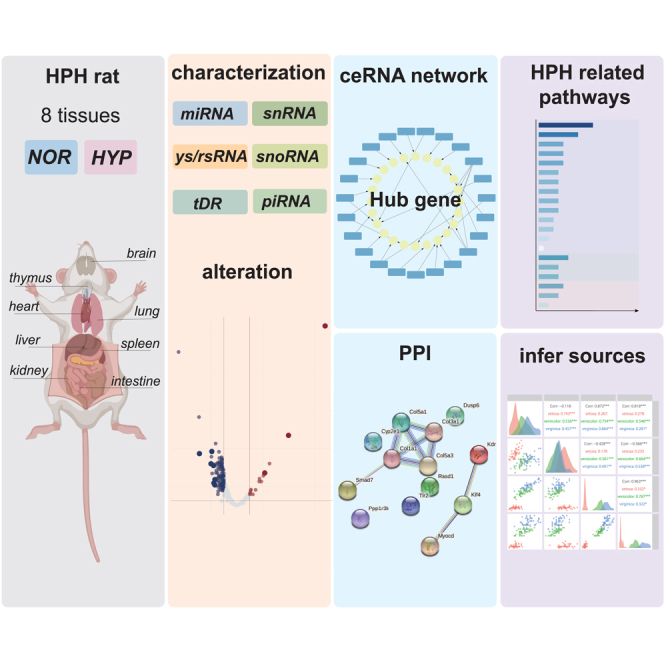

## Introduction

Hypoxia, a common pathological process in various human diseases,^1^^,^^2^^,^^3^ can cause histopathological abnormalities, metabolic dysregulation, physiological dysfunction, and mortality.^4^^,^^5^^,^^6^ Hypoxia-induced pulmonary hypertension (HPH) is a condition where diminished oxygen levels constrict pulmonary arteries, escalating pulmonary circulation resistance and pulmonary artery pressure.^7^ This constriction impedes pulmonary blood flow and increases the heart’s workload, leading to further tissue damage.

Small noncoding RNAs (sncRNAs) constitute a diverse class of endogenous transcripts, typically encompassing less than 300 nucleotides.^8^^,^^9^ Originating from various genomic loci, sncRNAs play essential roles in regulating cellular physiological activities, such as gene expression and cell proliferation.^8^^,^^10^^,^^11^^,^^12^^,^^13^^,^^14^ Differential expression patterns of distinct sncRNAs contribute to the dynamic molecular profiles observed in tissues or cells.^15^^,^^16^ Prominent examples of sncRNAs include ribosomal RNA-derived small RNA (rsRNA), RNY-derived small RNA (ysRNA), small nucleolar RNA (snoRNA), PIWI-interacting RNA (piRNA), small nuclear RNA (snRNA), microRNA (miRNA), and tRNA-derived small RNA (tDR) etc.^17^^,^^18^^,^^19^^,^^20^ In recent years, sncRNAs have attracted considerable interest owing to their implications in cellular regulation and association with diverse diseases.^14^^,^^21^^,^^22^

Studies have shown that miR-143, miR-20a, miR-125a-5p, miR-322, and miR-451 are upregulated, while miR-22, miR-30, and lef-7f are downregulated in lung tissues or pulmonary artery of HPH rats. Several miRNAs also contribute to HPH pathogenesis by impairing nitric oxide production, affecting smooth muscle cell proliferation and migration, reducing apoptosis, and promoting endothelial-to-mesenchymal transition in pulmonary arteries. Nevertheless, studies on the roles of other sncRNA types in HPH and their alteration in different tissues during HPH development remain limited.

To address this gap, we conducted a comprehensive investigation to characterize the impact of hypoxia on sncRNA profiles across eight different tissues in adult male HPH rats using high-throughput sequencing. This study provides an extensive and novel atlas of sncRNAs, including miRNA, snoRNA, snRNA, rsRNA, ysRNA, piRNA, and tDR, revealing tissue-specific and hypoxia-responsive sncRNAs, particularly within the repertoires of miRNA and tDR. Notably, hypoxia induced arm switching in several miRNAs and a significant increase in the proportion of 5′tR-halves among total tDRs. In addition, through characterizing the mRNA targets of differentially expressed miRNA (DEmiRNA) in the lung of HPH rats, a potential competitive endogenous RNA (ceRNA) regulatory network was constructed, which may be involved in the extracellular matrix and pulmonary artery remodeling during the progression of HPH. DEsncRNAs identified in this study were then validated through real-time quantitative reverse transcription PCR (qRT-PCR). In summary, our study presents a comprehensive characterization of sncRNA profiles in HPH rats, shedding light on the identification of sncRNAs involved in HPH-induced physiological dysfunction. This research has significant implications for advancing basic research and broadening the clinical applications of sncRNAs.

## Results

### Small noncoding RNA expression profile of rat tissues

**Figure 1:**
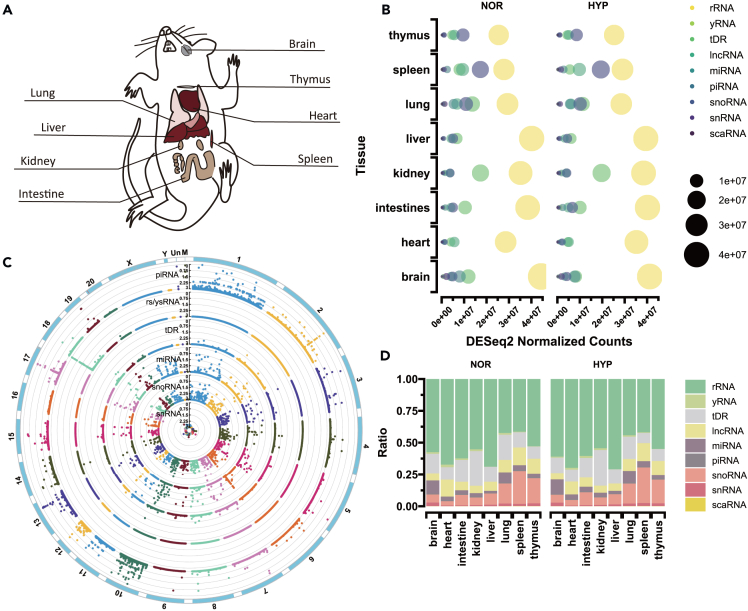
A rat tissue atlas of small noncoding RNA (sncRNA) in hypoxia and normoxia (A) Tissues characterized in this study: 8 tissues from adult male rat (n = 6, n_hypoxia_ = 3, n_normoxia_ = 3) were systematically profiled in our study. (B) sncRNA expression status in hypoxia and normoxia. Nine classes of RNA were quantified in our study, including rRNA, yRNA, tRNA, lncRNA, miRNA, piRNA, snoRNA, snRNA, as well as scaRNA. (C) The genomic map illustrates the expression pattern of sncRNAs across the rat genome under hypoxic conditions. (D) Proportion of each RNA species under normoxia or hypoxia.

To evaluate the sncRNA expression profile in HPH rats, we exposed healthy male Sprague-Dawley rats to chronic hypoxia for 21 days. After the hypoxia treatment, we measured the body weight (Figure S1A), right ventricular systolic pressure (RVSP) (Figure S1B), and right ventricular hypertrophy index (RVHI) of the rats (Figure S1C). Our findings revealed an increase in RVSP (28.88 ± 1.62 vs. 60.21 ± 2.91) and RVHI (0.20 ± 0.03 vs. 0.36 ± 0.03) in rats exposed to hypoxia. Furthermore, histological analysis of the lungs suggested pronounced pulmonary artery remodeling and increased pulmonary wall thickness in rats subjected to hypoxia (Figures S1D and S1E). These results indicate the successful construction of the HPH model. Different tissues (heart, liver, spleen, lung, kidney, brain, intestine, and thymus) from both HPH and normal rats were separated from connective tissue and cleaned for total RNA isolation, which was then subjected to sncRNA sequencing, respectively. We created a dataset consisting of 48 sequencing libraries for small noncoding RNAs, which were then used to analyze sncRNA expression within eight different tissues from adult male rats under both normoxia (n = 3) and hypoxia (n = 3) conditions (Figure 1A; Table S1). Each library was sequenced with a minimum of 30 million raw reads, with approximately 80% of these reads aligned to the rat genome. Our results showed that rsRNA was the most abundant type of annotated RNA, accounting for over 50% of the raw reads, which is consistent with rRNA’s pervasiveness in total RNA (Figure 1B). We then generated a genome-wide expression map of sncRNAs in rat tissues, revealing that sncRNAs (excluding rsRNAs and ysRNAs) were evenly distributed across the rat genome (Figure 1B). The proportion of different types of mapped sncRNAs varied across different tissues, with miRNAs, tDRs, and snoRNAs having the greatest abundance in the brain, kidney, and spleen, respectively (Figures 1B and 1D). However, there were no significant variations in the number of distinct sncRNAs identified between normoxia and hypoxia (Figure 1D).

### Tissue-specific small noncoding RNA expression

**Figure 2:**
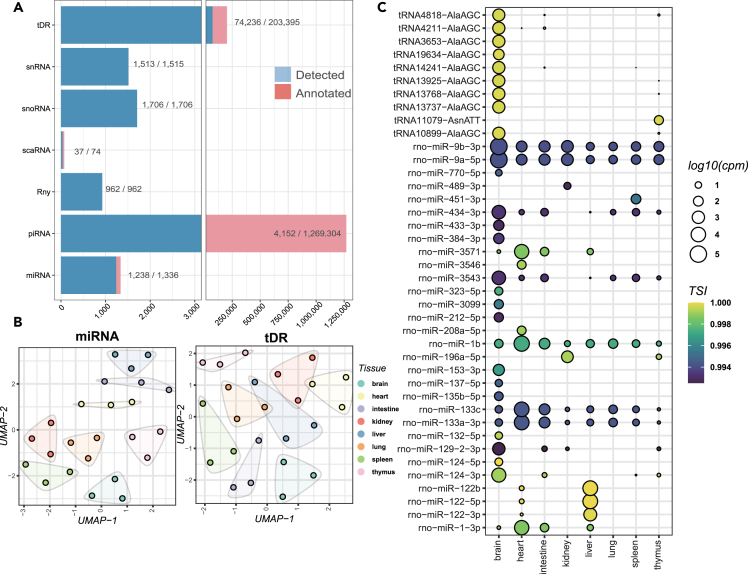
Expression patterns of small noncoding RNA (sncRNA) (A) All sncRNAs identified in current study. This study investigated the coverage of various sncRNA types across the analyzed tissue samples. In this study, a sncRNA was considered transcribed in a particular tissue if it was detected at a level greater than 1 count per million (cpm). (B) Expression patterns of sncRNAs: uniform manifold approximation and projection (U-MAP) was used to visualize the expression patterns of sncRNA genes across 8 different rat tissues. (C) Dot plot of examples of tissue-specific tDRs and miRNAs. The displayed RNAs achieved statistical significance (FDR <0.05) in the likelihood ratio test (LRT) conducted.

We quantified the expression of seven classes of sncRNAs, including ysRNAs, tDRs, miRNAs, piRNAs, snoRNAs, snRNAs, and scaRNAs across different tissues. In our dataset, we identified 74,236 tDRs, 1,238 miRNAs, 4,152 piRNAs, 1,706 snoRNAs, 1,513 snRNAs, and 37 scaRNAs, representing 36.5%, 92.7%, 0.3%, 100%, 99.9%, and 50% of annotated RNAs respectively (Figure 2A). Among these sncRNAs, tDRs were the most abundant, followed by piRNAs, snoRNAs, snRNAs, and miRNAs. Fragment length distribution analysis revealed no tissue specificity among these sncRNAs (Figure S2). We then evaluated the expression levels of sncRNAs across eight different tissues using variance-stabilizing transformed (VST) gene expression values. Dimension reduction analysis through principal component analysis (PCA) and uniform manifold approximation and projection (UMAP) analysis of all sncRNAs showed clear separation of tissues for tDRs and miRNAs under normoxia, suggesting their tissue specificity (Figures 2B and S2B). To further identify tissue-specific genes, differential analysis of all sncRNA classes was conducted, and the tissue specificity index (TSI) was further calculated. We found that 2,623 sncRNAs were expressed in a tissue-specific manner under normoxia, and the heart had the most sncRNAs with unique expression (Table S2).

Furthermore, miRNAs were found to be the main contributors to the tissue-specific expression signature in the dimension reduction analysis (Figure 2B). Out of the 214 DEmiRNAs, 114 were expressed specifically in the brain, 9 in the heart, 7 in the intestine, 7 in the kidney, 4 in the liver, 52 in the lung, 7 in the spleen, and 14 in the thymus (Table S1). For instance, rno-miR-212-5p was exclusively expressed in the brain, while rno-miR-196-5p was mainly expressed in the kidney and thymus (Figure 2C). The expression levels of rno-miR-434 and rno-miR-129 were increased in the brain but decreased in the intestine (Figure 2C). tDRs, as an indicator of transcriptional activity, also displayed tissue specificity. The expression of specific tDRs correlated with AlaAGC was observed exclusively in the brain. tRNA11079-AsnATT was observed to be expressed only in the thymus (Figure 2C). Some other sncRNAs also showed tissue specificity, such as Rny-262, Rny-626, Rny-1009, and Rny-1490, which were only detected in the kidney. A few piRNAs, such as piR-rno-161 and piR-rno-2023 in the thymus and piR-rno-4626 in the brain, also exhibited tissue-specific patterns (Table S3). Moreover, ENSRNOG00000057766, also known as SNORA70, was found to be specific to the heart (Table S3), similar to its primate orthologs.^23^

### Hypoxia-responsive small noncoding RNA expression

**Figure 3:**
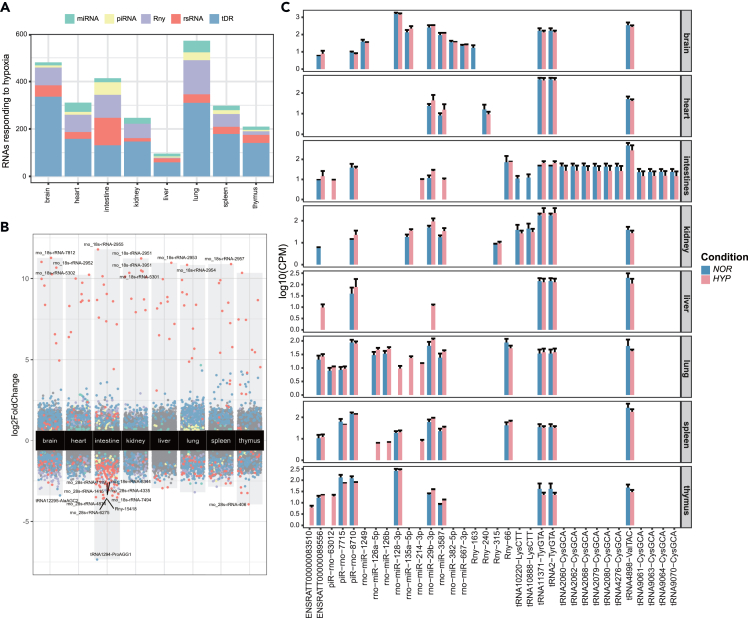
Hypoxia-specific expression patterns of small noncoding RNA (sncRNA) (A) Proportion of different classes of sncRNAs responding to hypoxia across 8 profiled tissues (FDR <0.05 and |log2FC| > 1). (B) Volcano plot of sncRNAs responding to hypoxia across 8 profiled tissues. (C) Hypoxia-specific miRNAs and tDRs identified in current study. The y axis represents the log10 of the difference between the mean miRNA levels in normal and hypoxia-induced pulmonary hypertension (HPH) rats for each tissue measured in counts per million (cpm). Data in (C) are expressed as mean ± standard deviation.

To evaluate the influence of hypoxia on the expression patterns of sncRNAs, we conducted t-distributed stochastic neighbor embedding (t-SNE) analysis on all sncRNA subclasses (Figures S3A and S3B). The output demonstrated a clear separation between hypoxic and normoxic tissues for miRNAs and tDRs, suggesting that these sncRNAs are also hypoxia-responsive. We detected various DEsncRNAs across tissues under hypoxic conditions (FDR <0.01, |log2FoldChange| > 2 and normalized read counts >10). The heart had the greatest number of hypoxia-induced DEsncRNAs (n = 491), followed by the intestine (n = 409), lung (n = 407), and brain (n = 377), while the liver had the lowest (Figures 3A and 3B; Table S2). To identify the sncRNAs that were responsive to hypoxia in different tissues, we fitted a generalized linear model to determine the relationship between sncRNA features and hypoxia. Ultimately, several sncRNAs were found to have both tissue specificity and the potential to be biomarkers for hypoxia in multiple organs (Figures 3A and 3B; Tables S4 and S5). Some of the DEsncRNAs were globally hypoxia-dependent across most tissues, while others showed tissue specificity. For example, the expression of rno-miR-29b-3p and rno-miR-3587 increased in most tissues under hypoxia, but the greatest increase was observed in the lung and intestine, respectively (Figure 3C; Tables S4 and S5). tRNA-4898-ValTAC was also found to be upregulated in most tissues under hypoxia, with the most significant up-regulation observed in the lung (Figure 3C; Tables S4 and S5). In contrast, the expression of piR-rno-8710 decreased in the brain and thymus under hypoxia but increased in the liver and kidney, remaining unchanged in the spleen and lung (Figure 3C; Table S4). Interestingly, tDRs correlated with AlaAGC were found to have both intestine specificity and a hypoxia-induced decreasing expression pattern (Figure 3C; Tables S4 and S5). Furthermore, we also discovered a hypoxia-sensitive genomic region consisting of six miRNAs, five of which were downregulated under hypoxia (Figure S3D).

### Novel microRNA identification

**Figure 4:**
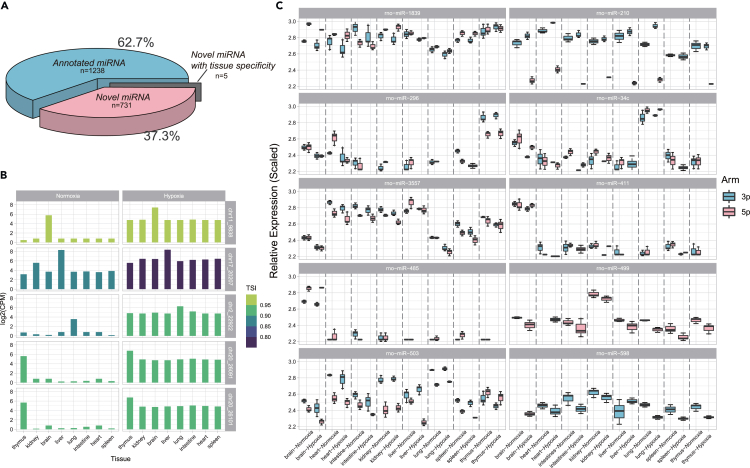
miRNAs uniquely detected in current study and tissue- and hypoxic-specific arm switch of miRNAs (A) Pie chart of novel and annotated miRNAs. (B) Novel miRNAs with expression in a tissue-specific pattern. (C) Hypoxic-specific arm switch of miRNAs. The graph depicts instances of arm switching in miRNAs under hypoxic conditions. Representative miRNAs consistently detected in either one or both arms, or with a switched arm between tissues, are included. The y axis reflects normalized scaled counts. Data in (C) are expressed as mean ± standard deviation.

The current miRbase annotation of mammals, such as rat, mouse, and human, has been demonstrated to be incomplete and can be expanded with the help of emerging high-throughput sequencing data.^24^ We employed miRDeep2^25^ in combination with high-throughput sequencing to identify a total of 736 novel miRNAs (Figure 4A; Table S6). We discovered that 731 out of the 1,969 miRNAs had been previously reported in other studies (Figure 4A). After filtering out miRNAs with expression levels lower than 10 counts in all 48 datasets, 357 miRNAs were left for further analysis. Notably, five of these novel miRNAs displayed tissue-specific expression patterns and typical pri-miRNA structures (Figures 4B and S4). For instance, chr11_9838 was exclusively expressed in the brain, chr2_2292 in the lung, and chr20_26091 and chr20_26101 in the thymus. Our results suggested that hypoxia treatment upregulated the expression of these novel miRNAs in various tissues, resulting in a lower TSI (Figure 4B). Most of these newly identified miRNAs were detected in the brain, while only a few were found in other tissues. Additionally, our findings suggest that these novel miRNAs can discriminate between tissues under normoxic and hypoxic conditions.

### Tissue- and hypoxic-specific arm switch of microRNAs

We evaluated the relative abundance of two distinct forms of miRNA, namely 3p and 5p, under normoxic and hypoxic conditions in the examined tissues. Our results indicated that the majority of miRNAs did not exhibit any significant differences (Figure S5). Most miRNAs showed a preference for either the 3p or 5p form, while some miRNAs displayed high expression levels for both forms in one or more tissues. Nevertheless, we also observed that roughly 3% of the miRNAs in our dataset had a change in the dominant arm among tissues or between different oxygen conditions (Figure 4C). For example, rno-miR-1839 had predominant expression of the -3p form in the intestine, but the expression of rno-miR-1839-5p was higher in other tissues. Similarly, rno-miR-296-5p was predominant in the heart, while the expression of rno-miR-296-3p was more prominent in the thymus. In some cases, only one arm of a miRNA was detected in normal tissue, but hypoxic treatment resulted in the detection of both arms. For instance, rno-miR-210-3p was detected in normal tissue, but both -3p and -5p were detected after hypoxic treatment (Figure 4C). Conversely, for rno-miR-499, only -5p was detected in normal tissue, but both arms were observed after hypoxic treatment (Figure 4C). Some miRNAs had equal expression levels of -3p and -5p in normal tissue but showed a distinct preference after hypoxic treatment. For example, rno-miR-503 had comparable expression levels in -3p and -5p in normal liver tissue, but -3p was significantly higher in hypoxic conditions (Figure 4C). On the other hand, miRNAs with clear arm selection in healthy tissue may exhibit comparable expression levels of both arms after hypoxic treatment. For instance, rno-miR-296 had a pronounced predominance of -5p expression in normal heart tissue, but after hypoxic treatment, the difference between the two arms was no longer observed (Figure 4C). Similarly, rno-miR-3557 exhibited significantly higher -5p expression in normal liver tissue compared to -3p, but after hypoxic treatment, the difference between the two arms was no longer existed (Figure 4C).

### Competitive endogenous RNA regulatory network construction and gene ontology analysis

**Figure 5:**
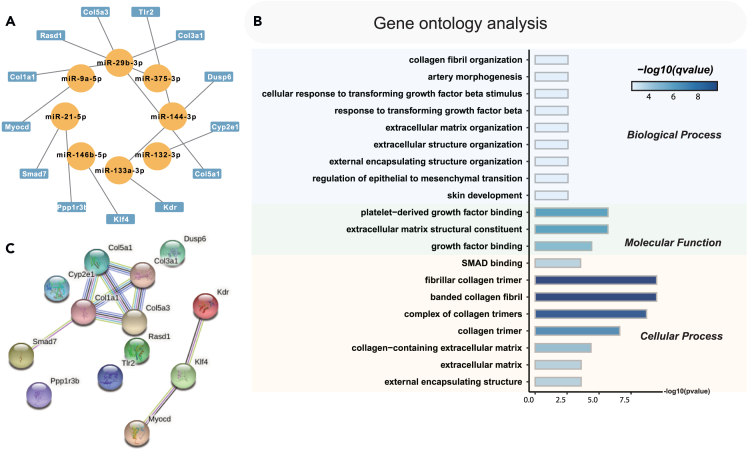
Potential competitive endogenous RNA (ceRNA) regulatory network and gene ontology (GO) analysis (A) A ceRNA regulatory network was systematically constructed in this study, involving 8 differentially expressed miRNAs (DEmiRNAs) and 13 DEmRNAs. (B) GO analysis of DEmRNA that targeted by DEmiRNA in the ceRNA regulatory network. Three aspects including biological process (BP), cellular component (CC), and molecular function (MF) were analyzed. (C) Results of protein-protein interactions (PPI) analysis of DEmRNA that targeted by DEmiRNA in the ceRNA regulatory network. The balls represent the gene nodes, the connecting lines represent the interactions between genes and figures insides the balls represent protein structure.

In order to gain further insights into the functional role of hypoxia-responsive miRNAs in the lungs of HPH rats, we aimed to investigate their potential mRNA targets. To accomplish this, we initiated RNA-seq analysis of lung tissues obtained from both HPH and normal rats, resulting in the identification of 2,746 differentially expressed mRNAs (DEmRNAs) (Table S7). Next, we explored the possible interactions between DEmiRNAs and DEmRNAs, and constructed a ceRNA regulatory network accordingly (Figure 5A). Remarkably, our findings revealed that 8 DEmiRNAs were capable of targeting a total of 13 DEmRNAs. Particularly, we observed that the upregulated rno-miR-29b-3p specifically targeted mRNAs including Col5a3, Col5a1, Col3a1, and Col1a1, thereby downregulating their expression (Figure 5A). Notably, these targeted DEmRNAs were primarily associated with extracellular matrix remodeling and artery morphogenesis, which are crucial processes involved in pulmonary artery remodeling during the progression of HPH (Figure 5B; Table S8). Furthermore, our protein-protein interactions (PPI) analysis of DEmRNAs targeted by DEmiRNAs in the ceRNA regulatory network indicates that Klf4 and Col1a1 function as central genes within the network (Figure 5C), underscoring the essential role of DEmiRNAs in mediating the lung’s response to hypoxia.

### Tissue- and hypoxic-specific tRNA-derived small RNAs

**Figure 6:**
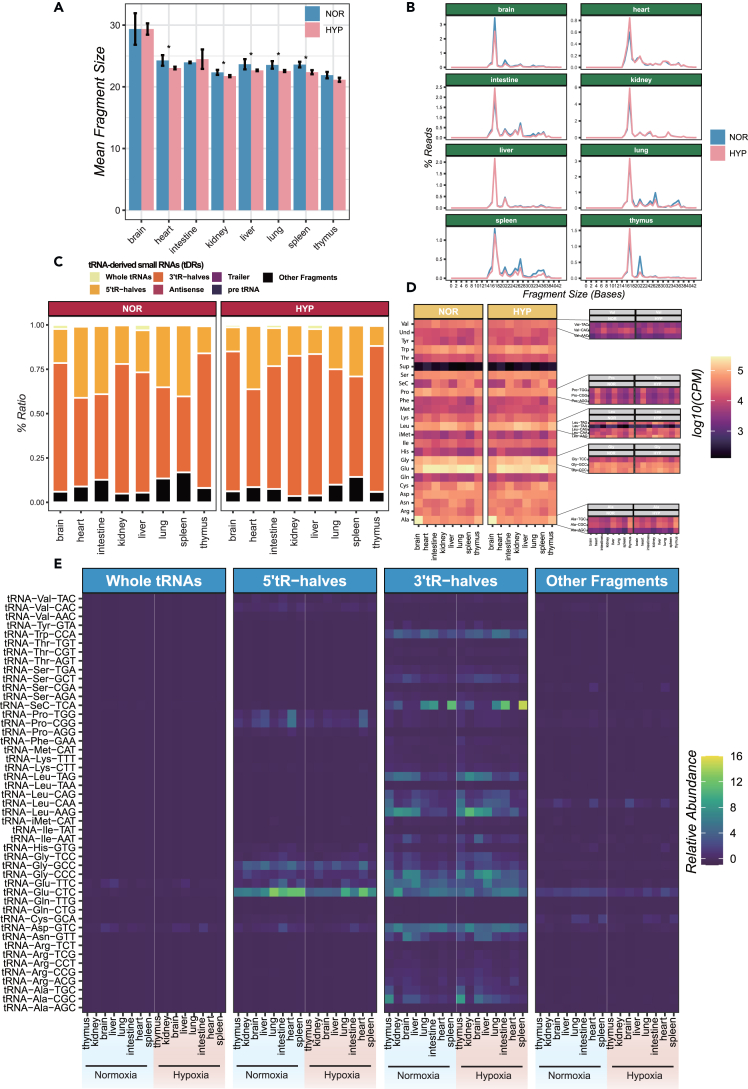
tRNA-derived small RNA (tDR) detected in current study (A) Changes in tDRs mean fragment size in normoxic and hypoxic conditions. (B) Length distribution of tDRs in normoxia and hypoxia. The length distribution of tDRs in normoxic and hypoxic conditions is depicted, providing insights into the average size of these fragments across various tissues. (C) Abundance of different fragment types in normoxic and hypoxic conditions across eight tissue samples. (D) Heatmap shows the expression levels of tDRs across different tissue samples. The sum of tDRs expression levels for each of the 23 tRNA types is plotted. (E) Heatmap displays the relative abundance of different types of tDR fragments across eight tissues, including whole tRNAs, 5′tR-halves, 3′tR-halves, and other fragments (such as trailer, antisense, pre tRNA, etc.). Row-wise scaled fractionated scores of tDRs, computed by tRNA analysis of eXpression (tRAX) software, represent the relative abundance for each tRNA isoacceptor. Data in (A) are expressed as mean ± standard deviation; ∗p < 0.05. Data are expressed as mean ± standard deviation.

We found that sequencing reads mapped to tDRs make up 10–20% of our sequencing data (Table S9), suggesting their crucial role in biological function. Most of these reads showed multiple mapping, which led us to hypothesize that tDRs have variable fragment sizes. Further analysis revealed that the fragment lengths of tDRs were significantly reduced or displayed a decreasing trend in most tissues under hypoxic conditions (Figure 6A; Table S9). This finding highlights the impact of hypoxia on tRNA cleavage and suggests potential roles for tDRs in hypoxia-induced gene regulation. An intriguing finding was the shift of the peak value of fragment size for tDRs in response to low oxygen conditions. Specifically, the proportion of tDR fragments ranging from 16 to 18 nt significantly increased under low-oxygen conditions (Figures 6A and 6B; Table S10), with the effect of being particularly prominent in the brain, lung, spleen, and thymus. To investigate whether global changes in tDR fragments contributed to the observed phenomenon, we analyzed the composition of tRNA fragment categories in the examined tissues. We found that the relative abundance of tRNA-derived fragments (tRF) types varies across different tissues. Notably, there was a significant increase in the proportion of 3′tR-halves among tRNA fragments under hypoxia (Figure 6C). We speculated that this increase might lead to changes in the tRNA fragment length distribution due to the smaller fragment size of 3′tR-havles. Furthermore, although tissue-specific differences were present across tRNA anticodons and isoacceptors, our results showed no significant changes caused by hypoxia (Figure 6D). While 3′tR-halves and 3′-tRFs were the dominant subtypes of tDRs under both normoxic and hypoxic conditions, we found that hypoxia might lead to a significant shift between each form. For instance, tRNA-SeC-TCA had more 3′tR-halves and shifted to a smaller size under low oxygen conditions, indicating its sensitivity to such condition (Figure 6E).

### Validation of differentially expressed small noncoding RNAs

**Figure 7:**
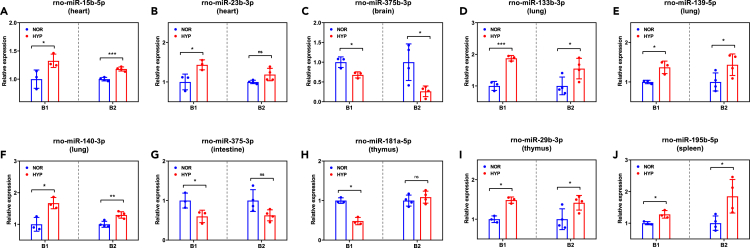
Expression profiles of selected differentially expressed small noncoding RNA (DEsncRNA) in different tissues of hypoxia-induced pulmonary hypertension (HPH) rats (A‒J) Expression level of rno-miR-15b-5p (A), rno-miR-23b-3p (B), rno-miR-375b-3p (C), rno-miR-133b-3p (D), rno-miR-139-5p (E), rno-miR-140-3p (F), rno-miR-375-3p (G), rno-miR-181a-5p (H), rno-miR-29b-3p (I), and rno-miR-195-5p (J) in different tissues of two batches (B1 and B2) of both normal and HPH rats. Data are expressed as mean ± standard deviation; ∗p < 0.05; ∗∗p < 0.01; ∗∗∗p < 0.001; ns, not significant; NOR, normal control rats; HYP, hypoxia-treated rats.

To validate the expression profiles of DEsncRNAs, the expression levels of selected DEsncRNAs from various tissues were assessed using qRT-PCR in both the current batch of HPH and normal rats and another independent batch of rats. With the exception of rno-miR-23b-3p from the heart, rno-miR-181a-5p from the thymus, and rno-miR-375-3p from the intestine, the remaining seven DEsncRNAs exhibited consistent expression patterns in both batches of HPH and normal rats, aligning with the sequencing results as expected (Figures 7A, 7C‒7F, 7I, and 7J). Although these three sncRNAs did not exhibit significant differential expression, their expression trends were in line with those observed in the initial batch and sequencing analysis (Figures 7B, 7G, and 7H). Overall, these validation results prove the reliability of the identified sncRNA expression profiles in this study.

## Discussion

sncRNAs play a crucial role in modulating essential cellular processes and influencing gene expression,^26^^,^^27^^,^^28^^,^^29^^,^^30^^,^^31^^,^^32^ thereby defining cellular identity and function in health and disease. In our study, we analyzed 48 sncRNA sequencing libraries from 8 different tissues (heart, liver, spleen, lung, kidney, brain, intestine, and thymus) of adult male HPH rats to investigate their tissue-specific and hypoxia-dependent expression patterns. Our analysis revealed the tissue-specific roles of sncRNAs in regulating gene expression and suggested their involvement in cellular responses to hypoxia across different organs.

Previous studies have shown that miRNAs, a well-studied class of sncRNAs, exhibit tissue- and cell type-specific expression patterns. Our work builds upon these findings by demonstrating that other classes of sncRNAs also display tissue- and hypoxia-responsive expression profiles. Through the analysis of sncRNA expression patterns in normal and HPH rat tissues, we generated a comprehensive sncRNA expression profile that unveiled the unique noncoding signatures of each tissue and provided valuable insights into the tissue-specific roles of sncRNAs in regulating gene expression under normoxic and hypoxic conditions.

We found that sncRNAs exhibit tissue-specific and hypoxia-responsive expression pattern, potentially influenced not only by transcription rates and posttranscriptional modifications, but also related to the selective RNA retention mechanism. For instance, certain miRNAs alter their arms between normoxic and hypoxic tissues, a phenomenon reported in various diseases.^33^^,^^34^ In our study, we also noted a selective distribution of certain fragment types of tDRs across specific genes and tissues under both normoxic and hypoxic conditions. These findings suggest that tDRs may have tissue-specific roles in regulating gene expression in response to hypoxia. In conjunction with previous studies, our results prompt further investigations into the biogenesis pathways and functional roles of tDRs in diverse tissues under hypoxia. Understanding the functions of tDRs may yield valuable insights into the mechanisms by which these molecules regulate gene expression and mediate cellular responses to hypoxia.^35^^,^^36^^,^^37^^,^^38^^,^^39^

As sncRNAs in blood have been demonstrated to have utility as biomarkers for various diseases. We also investigated their expression in the blood of HPH and normal rats. In our analysis, we identified 44 DEmiRNAs and 817 DEtDRs in the plasma of HPH (Figures S6A and S6B; Table S11). To decipher the origin of these DEmiRNAs, we conducted a correlation study comparing their fold changes in plasma with those in eight different tissues. Surprisingly, a significant correlation was observed between the DEmiRNAs identified in plasma and those in the thymus (Figure S6C). This observation led us to speculate that a substantial portion of the sncRNAs present in the plasma may originate from thymus-originating leukocytes, potentially accounting for the similar expression patterns between plasma and thymus in both HPH and normal rats. Our study identified several sncRNAs that are uniquely expressed in certain tissues but have not been reported in previous studies (Figure 2; Table S3). In particular, we found that miRNAs were the main contributors to tissue-specific expression signatures in our dimension reduction analysis. tRNAs were previously considered to be housekeeping genes due to their high cellular abundance and stability.^38^ However, recent evidence suggests that tRNAs are subject to tight regulation. Even small changes in tRNA abundance or nucleotide modification can have profound effects on cellular processes, leading to aberrant translation, changes in protein expression, and disease states. For instance, tRNA-AlaGCC has been found to be predominantly expressed in neurons and may play a crucial role in the development of neurological deficits,^40^ including altered motor coordination and aberrant exploratory behavior.^41^ Our findings support this observation, as we detected high expression levels of tRNA4818-AlaAGC exclusively in the brain (Tables S3 and S9).

miRNA, a type of sncRNA that has been extensively studied, has been shown to have a strong correlation with the onset and progression of HPH, for instance, miR-21 is an miRNA that is upregulated in HPH and can influence the behavior of pulmonary artery smooth muscle cells (PASMCs) and pulmonary artery endothelial cells (PAECs) by regulating genes such as PDCD4, PPARα, and RhoB,^42^ which promotes the development of HPH. miR-21 has also been found to be upregulated in heart tissue, which may be associated with right ventricular remodeling and dysfunction. Studies have found correlations between miR-21 expression and right ventricular function in patients with HPH and animal models.^43^ In our data, miR-21 was upregulated not only in the lung but also in the heart, indicating that miR-21 may play an important role in the development of HPH, particularly in right ventricular remodeling (Table S3). Therefore, miR-21 has the potential to serve as a diagnostic marker of HPH.

miR-210 is considered as a “master miRNA” that controls various cellular functions in the development of HPH.^44^ Research has shown that miR-210 can promote pulmonary hypertension by inhibiting apoptosis in PASMCs through targeting E2F3 and downregulating the expression of ISCU1/2 and COX10.^45^ In our data, miR-210 was upregulated not only in lung tissue but also in heart and brain tissue (Table S3). We speculate that this is because miR-210 is a marker of hypoxic stress response and may be associated with various ischemic diseases. It is upregulated in ischemic tissues and can regulate inflammation and fibrosis. The heart and brain are among the tissues most severely affected by hypoxia. Other HPH-related miRNA markers, such as miR-339,^46^ have also been validated to be downregulated in hypoxic lung tissue.

Additionally, we observed a clear separation between hypoxic and normoxic tissues for miRNAs and tDRs, indicating the hypoxia-responsive nature of these sncRNAs in HPH. The heart harbors the most diverse hypoxia-responsive sncRNAs. We found significant differences in the expression of miRNAs and tDRs, two classes of sncRNAs that regulate gene expression and translation, respectively. These differences indicate that these sncRNAs are responsive to hypoxia and may play an important role in HPH development. Among the DEsncRNAs, some were consistently altered by hypoxia in all tissues, while others showed tissue-specific changes. For instance, miR-29b-3p was upregulated in most investigated tissues under hypoxia, this finding is consistent with previous reports on the miR-29 families.^47^ Previous studies have reported that miR-29a^48^ and miR-29c^49^ are upregulated in response to hypoxia. However, the relationship between miR-29b and hypoxia is not yet fully understood. Despite this, there is evidence suggesting that miR-29b is associated with several hypoxia-related diseases and may have potential as a biomarker for these conditions in multiple systems. It has been demonstrated that miR-29b-3p may activate the MIR497HG/miR-29b-3p/SIRT1 axis or inhibit Bcl2L2 to counteract neuroinflammation in the brain.^50^

Furthermore, it was reported that miR-29b-3p increased in cardiomyocytes under hypoxic stress and may protect them from apoptosis by targeting TRAF5.^51^ Our data support these findings and also show that miR-29b-3p increased under hypoxic stress in various tissues, suggesting its potential as a biomarker for cellular hypoxia in multiple hypoxia-related diseases. Unlike miR-29b-3p, our data showed that miR-3587 and tRNA-4898-ValTAC also increased under hypoxia in most tissues, with the highest levels in the intestine and lung, respectively. These findings imply that these molecules may have a role in HPH development. (Figures 3 and 4; Table S3).

We have successfully identified several DEmiRNAs implicated in HPH development within the lung and heart tissues. These findings suggest a potential involvement of these miRNAs in the pathogenesis of HPH. However, the significance of DEmiRNAs identified in tissues beyond the lung and heart in relation to HPH remains largely unexplored. For instance, given the substantial role of altered immune mechanisms in PAH development, characterized by the recruitment of inflammatory cells and remodeling of the pulmonary vasculature, it is imperative to investigate whether DEmiRNAs identified in tissues such as the thymus have an impact on the immune response. Notably, T and B lymphocytes, pivotal effectors in the immune response integral to PAH progression, undergo selection in the thymus and bone marrow, respectively.^52^^,^^53^ Therefore, further investigations are needed to elucidate whether the identified DEmiRNAs in the thymus influence immune responses, subsequently affecting the development of PAH. This exploration will enhance our understanding of the intricate molecular mechanisms underlying PAH pathology beyond the traditionally studied lung and heart tissues.

Our study illuminates the tissue-specific and hypoxia-responsive properties of sncRNAs in adult HPH rats. The results provide a comprehensive atlas of sncRNA tissue identity, which may serve as a valuable resource for both basic and clinical research. Future research could build on our findings by exploring the molecular mechanisms through which sncRNAs regulate gene expression in different tissues under normoxic and hypoxic conditions. This could enhance our understanding of the functional roles of sncRNAs in gene regulation.

### Limitation of the study

Our study has identified a subset of sncRNAs that exhibit tissue-specificity and responsiveness to hypoxic conditions in HPH rats. Despite this significant finding, the precise mechanisms underlying the observed alterations in these DEsncRNAs remain elusive. Furthermore, the functional implications of these DEsncRNAs in relation to lung function and right ventricular pressure during the development of HPH are not fully understood. While some of the DEsncRNAs have been independently validated in a separate cohort of HPH rats, it is noteworthy that the initial profiling study, involving a limited sample size of 3 normal rats and 3 HPH rats, may not fully capture the heterogeneity of the HPH rat population. Consequently, there is a need for future research to expand upon these findings by employing qRT-PCR to assess the expression levels of DEsncRNAs in a more extensive cohort of HPH rats. In conclusion, subsequent investigations with larger and more diverse cohorts, coupled with mechanistic studies, will contribute to a more comprehensive understanding of the role of sncRNAs in the pathogenesis of HPH.

## STAR★Methods

### Key resources table

**Table:** 

REAGENT or RESOURCE	SOURCE	IDENTIFIER
**Chemicals, peptides, and recombinant proteins**		

SYBR	ThermoFisher	S33102

**Critical commercial assays**		

RNAiso Plus	Takara	9108
*E. coli* Poly(A) Polymerase	New England Biolabs	M0276L
Super M-MuLV Reverse Transcriptase	Fapon Biotech	MD028
AMpure XP beads	Beckman Coulter	A63881

**Deposited data**		

Raw data of NGS	This paper	SRA: PRJNA967621

**Experimental models: Organisms/strains**		

Sprague-Dawley (SD) rats	Guangdong Medical Laboratory Animal Center	N/A

**Oligonucleotides**		

qPCR primer, see Table S12	This paper	N/A

**Software and algorithms**		

fastp	https://github.com/OpenGene/fastp	N/A
cutadapt	https://github.com/marcelm/cutadapt	N/A
bowtie2	https://github.com/BenLangmead/bowtie2	N/A
AcqKnowledge	https://www.biopac.com/	N/A
subread	https://subread.sourceforge.net/featureCounts.html	N/A
tRAX	http://trna.ucsc.edu/tRAX/	N/A
DEseq2	https://bioconductor.org/packages/release/bioc/html/DESeq2.html	N/A
limma	https://bioconductor.org/packages/release/bioc/html/limma.html	N/A
miRDeep2	https://github.com/rajewsky-lab/mirdeep2	N/A
trimmomatic	https://github.com/usadellab/Trimmomatic	N/A
R	https://www.r-project.org/	N/A
tidyverse	https://github.com/tidyverse/tidyverse	N/A
ggplot2	https://github.com/tidyverse/ggplot2	N/A
GraphPad Prism 9	GraphPad	N/A
python	https://www.python.org/	N/A
SPSS 25.0	http://www.spss.com.cn	N/A

### Resource availability

#### Lead contact

Further information and requests for reagents and resources should be directed to and will be fulfilled by the lead contact, Deming Gou (dmgou@szu.edu.cn).

#### Materials availability

This study did not generate new unique reagents.

#### Data and code availability

• RNA-seq data have been deposited at Sequence Read Archive (SRA) and are publicly available as of the date of publication. Accession number is listed in the key resources table.
• All original code has been deposited at github and is publicly available as of the data of publication (https://github.com/Jiahao-Kuang/Characterization_of_Small-ncRNA_Profiles_in_HPH_Rat).
• Any additional information required to reanalyze the data reported in this paper is available from the lead contact upon request.

### Experimental model and study participant details

Healthy male Sprague-Dawley (SD) rats (8-week-old) utilized in this study were procured from the Guangdong Medical Laboratory Animal Center (Guangzhou, China). All experiments followed approved protocols from the animal care committee of Shenzhen University, China.

### Method details

#### Construction of HPH rat model and sample collection

Healthy male Sprague-Dawley (SD) rats (8-week-old) were randomly divided into normoxia and hypoxia groups with 3 or 4 rats in each group. Rats were exposed to normoxic (21% O2) or hypoxic (10% O2) conditions for 3 weeks respectively. Oxygen concentrations were monitored by detecting probes inside the chambers. We measured right ventricular pressure (RVSP), right ventricular hypertrophy index (RVHI) and relative wall thickness to evaluate the effect of hypoxia on the rats. We anesthetized the rats with 65 mg/kg pentobarbital sodium by intraperitoneal injection. The right jugular vein was then surgically exposed, and a polyethylene catheter connected to an AP-621G (Nihon Kohden, Japan) was inserted into the right ventricle (RV) to record RVSP using an MP150 system and AcqKnowledge 4.2.0 software package (BIOPAC Systems, USA). The right ventricle (RV) was weighed after separating it from the left ventricle (LV) and the ventricular septum (S). The ratio of RV weight to the sum of LV and S weights (RV/(LV + S)) was calculated as an index of RV hypertrophy (RVHI). The left lung lobes were fixed in formalin and stained with hematoxylin and eosin (HE). To quantify the medial wall thickness, 20–30 pulmonary arteries, with diameter of 50–100 μm, were inspected from each rat. The percentage of wall thickness was calculated using the following formula: relative wall thickness = (outer perimeter - inside perimeter)/outer perimeter. Tissues from the heart, liver, spleen, lung, kidney, brain, intestines, and thymus were collected and snap-frozen in liquid nitrogen for RNA extraction and sequencing library construction. For all the rats, 2 mL of whole blood samples were collected in EDTA anticoagulated vacutainers. Plasma separation was conducted within 3 h of whole blood collection, and the plasma was split into 500ul aliquots and stored at −80°C.

#### sncRNA sequencing library preparation

To isolate total RNA from the eight different tissues and plasma, we used RNAiso Plus (Takara, Japan) and Apostle MiniMaxTM High Efficiency cfRNA Isolation Kit (Apostle) according to the manufacturer’s instructions. Quality control measures were taken to ensure the RNA was suitable for downstream applications. The concentration of RNA was measured using a NanoDrop 2000c spectrophotometer (Thermo Fisher Scientific, USA), DNA contamination was assessed by gel electrophoresis (EPS 601, GE Healthcare, USA), and RNA integrity was evaluated using an Agilent 2100 bioanalyzer (Agilent, USA).

For characterizing sncRNA expression, we constructed sequencing libraries using the S-Poly (T) method described in a previous study, with some modifications.^54^ In brief, we used 500 ng of qualified total RNA extracted from the eight different tissues and all the RNA extracted from the plasma as the starting material. One-step poly-adenylation and reverse transcription (Poly(A)/RT) were performed using 5 μL of 4× reaction buffer, 1 μL 2.5 μlM RT primer, and 1 μL Poly(A)/RT enzyme, and the reaction was incubated at 37°C for 30 min. The resulting first-strand cDNA was then ligated to a splint adapter with a random single-stranded overhang and ligation blocking modification, as reported previously.^55^ The cDNA was amplified to generate sncRNA sequencing library, and the library was purified using AMpure XP beads (Beckman, USA) to select DNA fragments with an average size of approximately 180–200 bps. Finally, the libraries were subjected for bioanlyzer analysis before sequencing on illumina Novaseq platform at HaploX (Shenzhen, China) with 2✕150bp paired-en (PE) reads.

#### Data processing and data visualization

We conducted a thorough quality control check on the raw sequencing data using fastp, which included an assessment of the sequencing quality, splice junctions at each base in the sequence, and percentage distribution of GC content. The adapter and poly-A sequences at the 3′ end of R1 raw sequencing reads were cut away using Cutadapt. Low quality reads (q < 20) and reads less than 15 nt in length in the R1 file were trimmed away using Trimmomatic. The trimmed clean R1 reads were then aligned to the full length ribosomal RNA (rRNA; 28S, 18S, 5.8S, 5S, mt-16S and mt-12S) and Y RNA (RNY1, RNY3, RNY4 and RNY5) sequences obtained from NCBI using Bowtie2. Small RNA fragments mapped to rRNA and Y RNA were considered as rsRNA and ysRNA, respectively. The remaining R1 reads that unmapped to rRNA or Y RNA were then underwent alignment to the rat genome (Rnor_6.0) using Bowtie2. We removed the reads that aligned to mRNA and utilized annotation files obtained from various databases (Ensembl, miRbase, TANRIC, piRBase) to quantify snoRNAs, snRNAs, miRNAs, piRNAs, and lncRNAs using featureCounts. We used tRNA Analysis of eXpression (tRAX) software to quantify expression of tRNAs. All bioinformatics analyses were performed using R Version 4.2, and the visualizations were generated using publicly available packages such as ggplot2 and CMplot.

#### Analysis of unsupervised clustering and dimensionality reduction

We normalized the raw count values in all data using the DESeq2 package and obtained VST values for each gene. We applied the limma package to eliminate potential batch effects. Furthermore, we calculated Euclidean distances between each sample to generate UMAP results, which were conducted using the umap package with parameters set to random_state = 123, n_neighbors = 15, min_dist = 0.5, and spread = 0.9. We only included genes with CPM >15 in at least two samples for the analysis.

#### Identification of novel miRNAs

To identify candidate miRNAs, we utilized the miRDeep2 software. First, we filtered the mapping results of clean reads obtained from upstream processing to retain only reads that were perfectly aligned to the genome with length between 18 and 25 bp. We also removed reads with more than five hits to the genome. Candidate miRNAs predicted by miRDeep2 were considered novel miRNAs only if they met the following criteria: p < 0.05 and a count number >10 in at least two samples.

#### ceRNA network construction and gene function annotation

DEmiRNA (|Log2FoldChange| > 0.5 and p < 0.05) and DEmRNA (|Log2FoldChange| > 0.5 and p < 0.05) were identified by DEseq2 (v.1.32.0). Targeting relationships between DEmiRNAs–DEmRNAs were predicted mainly based on miRcode (http://mircode.org/) and TargetScan (http://www.targetscan.org/). The selected DEmiRNAs were then used as hub genes to build the ceRNA regulatory network in Cytoscape (v3.8.2). GO analysis was conducted based on DEmRNAs that targeted by the DEmiRNAs to evaluate enrichment for biological process (BP), cellular component (CC), and molecular function (MF) annotations with clusterProfiler (v4.0.0) R package. KEGG analysis was also performed to enrich the signaling pathways using clusterProfiler (v4.0.0) R package. GO terms and KEGG pathways with enriched genes >2 and p < 0.05 were selected for further analysis. The top 20 ranked GO terms and KEGG pathways containing most genes were visualized by ggplot2 (v3.3.3) R package.

#### Quantitative real-time PCR

To evaluate sncRNA expression profiles, first strand cDNA was reverse transcribed with oligo (dT) using M-MuLV reverse transcriptase (FAPON, China). Quantitative real-time PCR was conducted on ABI 7500 real-time PCR system (Applied Biosystems, USA) with SYBR green master PCR mix and gene-specific forward primer and universal reverse primer. Expression levels of targeted genes were normalized by the reference gene (snoRNA234). Relative expression of all RNAs was calculated according to the 2 ^-△△Ct^ method. All primers used in this study were listed in Table S12.

### Quantification and statistical analysis

We used SPSS software for statistical analysis. We checked the normality and homogeneity of variance of the data using Shapiro-Wilk test and Levene tests respectively. We used the Student’s *t* test to compare the means or medians of RVSP, RVHI, and wall thickness between hypoxia and normoxia groups. We used DESeq2 package in R to perform differential gene expression analysis between hypoxia and normoxia groups in different tissues. We used a false discovery rate (FDR) of 0.05 as the threshold for significance.

To determine the expression of sncRNAs associated with different tissues, we utilized the R package DESeq2. Briefly, we first performed TSS normalization on the expression data and subsequently applied AST transformation to the normalized data. We then employed a multivariate linear regression model provided by DESeq2 to identify sncRNAs related to tissues. Furthermore, we calculated the tissue-specific index (TSI) of these sncRNAs using the formula previously described.^56^ RNAs with TS1 > 0.85 were considered tissue-specific.

$${TSI}_{j}=\frac{\sum_{i=1}^{N}\left(1-x_{j,i}\right)}{N-1}$$

N represent the total number of tissues measured, and x_j,i_ denotes the expression score of tissue i, normalized by the maximal expression observed across all tissues for miRNA j.^56^

To investigate whether sncRNAs correlated with tissue expression would change under hypoxia, we conducted a differential analysis for each tissue separately using the DESeq2 package. We established the following criteria for significance: adjusted p values less than 0.05 and an absolute log2 fold change greater than 1. We then fitted a generalized linear model to determine the relationship between these sncRNA features and HPH. Ultimately, sncRNAs with p values less than 0.001 and adjusted p values less than 0.01 were considered to have both tissue specificity and the potential to serve as HPH biomarkers in multiple organs.

## Supplemental information

Document S1. Figures S1‒S6

Table S1. Expression set of small noncoding RNAs (sncRNAs) generated in current study, related to Figure 1

Table S2. Number of small noncoding RNAs (sncRNAs) with unique expression, related to Figure 2

Table S3. Tissue specificity of small noncoding RNAs (sncRNAs): normalized counts and tissue specificity index (TSI), related to Figure 2

Table S4. Differential analysis results in hypoxia-induced pulmonary hypertension (HPH) rats, related to Figure 3

Table S5. Significant differentially expressed small noncoding RNAs (DEsncRNAs) in each tissue, related to Figure 3

Table S6. Novel miRNAs detected by miRDeep2, related to Figure 4

Table S7. Differential analysis of mRNA in lung of hypoxia-induced pulmonary hypertension (HPH) rats, related to Figure 5

Table S8. Gene ontology (GO) analysis of differentially expressed mRNA (DEmRNA), related to Figure 5

Table S9. Expression of each type of tRNA-derived small RNAs (tDRs), related to Figure 6

Table S10. Length distribution of tRNA-derived small RNAs (tDRs), related to Figure 6

Table S11. Differential analysis of small noncoding RNAs (sncRNAs) in plasma, related to Figures 5 and S8

Table S12. Oligos used in this study, related to Figure 7

## References

[1] Lee J.W., Ko J., Ju C., Eltzschig H.K. (2019). Hypoxia signaling in human diseases and therapeutic targets. Exp. Mol. Med..

[2] Taylor C.T., Colgan S.P. (2017). Regulation of immunity and inflammation by hypoxia in immunological niches. Nat. Rev. Immunol..

[3] Tirpe A.A., Gulei D., Ciortea S.M., Crivii C., Berindan-Neagoe I. (2019). Hypoxia: Overview on Hypoxia-Mediated Mechanisms with a Focus on the Role of HIF Genes. Int. J. Mol. Sci..

[4] MacIntyre N.R. (2014). Tissue hypoxia: implications for the respiratory clinician. Respir. Care.

[5] Fajersztajn L., Veras M.M. (2017). Hypoxia: From Placental Development to Fetal Programming. Birth Defects Res..

[6] Campos J.H., Feider A. (2018). Hypoxia During One-Lung Ventilation-A Review and Update. J. Cardiothorac. Vasc. Anesth..

[7] Humbert M., Montani D., Evgenov O.V., Simonneau G. (2013). Definition and classification of pulmonary hypertension. Handb. Exp. Pharmacol..

[8] Cech T.R., Steitz J.A. (2014). The noncoding RNA revolution-trashing old rules to forge new ones. Cell.

[9] Choudhuri S. (2010). Small noncoding RNAs: biogenesis, function, and emerging significance in toxicology. J. Biochem. Mol. Toxicol..

[10] Agrawal N., Dasaradhi P.V.N., Mohmmed A., Malhotra P., Bhatnagar R.K., Mukherjee S.K. (2003). RNA interference: biology, mechanism, and applications. Microbiol. Mol. Biol. Rev..

[11] Bartel D.P. (2018). Metazoan MicroRNAs. Cell.

[12] Shi J., Zhou T., Chen Q. (2022). Exploring the expanding universe of small RNAs. Nat. Cell Biol..

[13] Huang C.Y., Wang H., Hu P., Hamby R., Jin H. (2019). Small RNAs - Big Players in Plant-Microbe Interactions. Cell Host Microbe.

[14] Desgranges E., Marzi S., Moreau K., Romby P., Caldelari I. (2019). Noncoding RNA. Microbiol. Spectr..

[15] Sun B.K., Tsao H. (2008). Small RNAs in development and disease. J. Am. Acad. Dermatol..

[16] Huang Y., Zhang J.L., Yu X.L., Xu T.S., Wang Z.B., Cheng X.C. (2013). Molecular functions of small regulatory noncoding RNA. Biochemistry.

[17] Romano G., Veneziano D., Acunzo M., Croce C.M. (2017). Small non-coding RNA and cancer. Carcinogenesis.

[18] Shi J., Zhang Y., Tan D., Zhang X., Yan M., Zhang Y., Franklin R., Shahbazi M., Mackinlay K., Liu S. (2021). PANDORA-seq expands the repertoire of regulatory small RNAs by overcoming RNA modifications. Nat. Cell Biol..

[19] Childs-Disney J.L., Yang X., Gibaut Q.M.R., Tong Y., Batey R.T., Disney M.D. (2022). Targeting RNA structures with small molecules. Nat. Rev. Drug Discov..

[20] Fabian M.R., Sonenberg N., Filipowicz W. (2010). Regulation of mRNA translation and stability by microRNAs. Annu. Rev. Biochem..

[21] Zhang L., Lu Q., Chang C. (2020). Epigenetics in Health and Disease. Adv. Exp. Med. Biol..

[22] Shah A.M., Giacca M. (2022). Small non-coding RNA therapeutics for cardiovascular disease. Eur. Heart J..

[23] Isakova A., Fehlmann T., Keller A., Quake S.R. (2020). A mouse tissue atlas of small noncoding RNA. Proc. Natl. Acad. Sci. USA.

[24] Alles J., Fehlmann T., Fischer U., Backes C., Galata V., Minet M., Hart M., Abu-Halima M., Grässer F.A., Lenhof H.P. (2019). An estimate of the total number of true human miRNAs. Nucleic Acids Res..

[25] Friedländer M.R., Mackowiak S.D., Li N., Chen W., Rajewsky N. (2012). miRDeep2 accurately identifies known and hundreds of novel microRNA genes in seven animal clades. Nucleic Acids Res..

[26] Cai Y., Yu X., Hu S., Yu J. (2009). A brief review on the mechanisms of miRNA regulation. Dev. Reprod. Biol..

[27] Saliminejad K., Khorram Khorshid H.R., Soleymani Fard S., Ghaffari S.H. (2019). An overview of microRNAs: Biology, functions, therapeutics, and analysis methods. J. Cell. Physiol..

[28] Lu T.X., Rothenberg M.E. (2018). MicroRNA. J. Allergy Clin. Immunol..

[29] Vishnoi A., Rani S. (2017). MiRNA Biogenesis and Regulation of Diseases: An Overview. Methods Mol. Biol..

[30] Krol J., Loedige I., Filipowicz W. (2010). The widespread regulation of microRNA biogenesis, function and decay. Nat. Rev. Genet..

[31] Snyder M.W., Kircher M., Hill A.J., Daza R.M., Shendure J. (2016). Cell-free DNA Comprises an In Vivo Nucleosome Footprint that Informs Its Tissues-Of-Origin. Cell.

[32] Hill M., Tran N. (2021). miRNA interplay: mechanisms and consequences in cancer. Dis. Model. Mech..

[33] Kern F., Amand J., Senatorov I., Isakova A., Backes C., Meese E., Keller A., Fehlmann T. (2020). miRSwitch: detecting microRNA arm shift and switch events. Nucleic Acids Res..

[34] Chen L., Sun H., Wang C., Yang Y., Zhang M., Wong G. (2018). miRNA arm switching identifies novel tumour biomarkers. EBioMedicine.

[35] Cunningham F., Allen J.E., Allen J., Alvarez-Jarreta J., Amode M.R., Armean I.M., Austine-Orimoloye O., Azov A.G., Barnes I., Bennett R. (2022). Ensembl 2022. Nucleic Acids Res..

[36] Orellana E.A., Siegal E., Gregory R.I. (2022). tRNA dysregulation and disease. Nat. Rev. Genet..

[37] Huang J., Chen W., Zhou F., Pang Z., Wang L., Pan T., Wang X. (2021). Tissue-specific reprogramming of host tRNA transcriptome by the microbiome. Genome Res..

[38] Pinkard O., McFarland S., Sweet T., Coller J. (2020). Quantitative tRNA-sequencing uncovers metazoan tissue-specific tRNA regulation. Nat. Commun..

[39] Baymiller M., Nordick B., Forsyth C.M., Martinis S.A. (2022). Tissue-specific alternative splicing separates the catalytic and cell signaling functions of human leucyl-tRNA synthetase. J. Biol. Chem..

[40] Gao W., Gallardo-Dodd C.J., Kutter C. (2022). Cell type-specific analysis by single-cell profiling identifies a stable mammalian tRNA-mRNA interface and increased translation efficiency in neurons. Genome Res..

[41] Jonkhout N., Cruciani S., Santos Vieira H.G., Tran J., Liu H., Liu G., Pickford R., Kaczorowski D., Franco G.R., Vauti F. (2021). Subcellular relocalization and nuclear redistribution of the RNA methyltransferases TRMT1 and TRMT1L upon neuronal activation. RNA Biol..

[42] Sarkar J., Gou D., Turaka P., Viktorova E., Ramchandran R., Raj J.U. (2010). MicroRNA-21 plays a role in hypoxia-mediated pulmonary artery smooth muscle cell proliferation and migration. Am. J. Physiol. Lung Cell Mol. Physiol..

[43] Boucherat O., Potus F., Bonnet S. (2015). microRNA and Pulmonary Hypertension. Adv. Exp. Med. Biol..

[44] Chan S.Y., Loscalzo J. (2010). MicroRNA-210: a unique and pleiotropic hypoxamir. Cell Cycle.

[45] Chan S.Y., Zhang Y.-Y., Hemann C., Mahoney C.E., Zweier J.L., Loscalzo J. (2009). MicroRNA-210 controls mitochondrial metabolism during hypoxia by repressing the iron-sulfur cluster assembly proteins ISCU1/2. Cell Metab..

[46] Chen J., Cui X., Li L., Qu J., Raj J.U., Gou D. (2017). MiR-339 inhibits proliferation of pulmonary artery smooth muscle cell by targeting FGF signaling. Physiol. Rep..

[47] Heid J., Cencioni C., Ripa R., Baumgart M., Atlante S., Milano G., Scopece A., Kuenne C., Guenther S., Azzimato V. (2017). Age-dependent increase of oxidative stress regulates microRNA-29 family preserving cardiac health. Sci. Rep..

[48] Wang Y., Qiu Z., Yuan J., Li C., Zhao R., Liu W., Deng W., Gu N., Zhang W., Hu S. (2021). Hypoxia-reoxygenation induces macrophage polarization and causes the release of exosomal miR-29a to mediate cardiomyocyte pyroptosis. In Vitro Cell. Dev. Biol. Anim..

[49] Fang Y., Yu X., Liu Y., Kriegel A.J., Heng Y., Xu X., Liang M., Ding X. (2013). miR-29c is downregulated in renal interstitial fibrosis in humans and rats and restored by HIF-α activation. Am. J. Physiol. Renal Physiol..

[50] Shi G., Liu Y., Liu T., Yan W., Liu X., Wang Y., Shi J., Jia L. (2012). Upregulated miR-29b promotes neuronal cell death by inhibiting Bcl2L2 after ischemic brain injury. Exp. Brain Res..

[51] Cai Y., Li Y. (2019). Upregulation of miR-29b-3p protects cardiomyocytes from hypoxia-induced apoptosis by targeting TRAF5. Cell. Mol. Biol. Lett..

[52] Huertas A., Perros F., Tu L., Cohen-Kaminsky S., Montani D., Dorfmüller P., Guignabert C., Humbert M. (2014). Immune dysregulation and endothelial dysfunction in pulmonary arterial hypertension: a complex interplay. Circulation.

[53] Tomaszewski M., Bębnowska D., Hrynkiewicz R., Dworzyński J., Niedźwiedzka-Rystwej P., Kopeć G., Grywalska E. (2021). Role of the Immune System Elements in Pulmonary Arterial Hypertension. J. Clin. Med..

[54] Niu Y., Zhang L., Qiu H., Wu Y., Wang Z., Zai Y., Liu L., Qu J., Kang K., Gou D. (2015). An improved method for detecting circulating microRNAs with S-Poly(T) Plus real-time PCR. Sci. Rep..

[55] Raine A., Manlig E., Wahlberg P., Syvänen A.C., Nordlund J. (2017). SPlinted Ligation Adapter Tagging (SPLAT), a novel library preparation method for whole genome bisulphite sequencing. Nucleic Acids Res..

[56] Ludwig N., Leidinger P., Becker K., Backes C., Fehlmann T., Pallasch C., Rheinheimer S., Meder B., Stähler C., Meese E., Keller A. (2016). Distribution of miRNA expression across human tissues. Nucleic Acids Res..

