# Supplementary material for: Comprehensive characterization of small noncoding RNA profiles in hypoxia-induced pulmonary hypertension (HPH) rat tissues
Source: iScience. 2024 Jan 13;27(2):108815. doi: 10.1016/j.isci.2024.108815 (PMC10844824; doi:10.1016/j.isci.2024.108815)

iScience, Volume *27*

Supplemental information

Comprehensive characterization of small noncoding RNA profiles in hypoxia-induced pulmonary hypertension (HPH) rat tissues

Jun Wang, Jiahao Kuang, Shasha Zhang, Zixin Liu, Qianwen Guo, Shujin Li, Lin Qiu, Gaohui Fu, Xinyang Lin, Jiayu Wu, Jinglin Tian, Jinyong Huang, Yanqin Niu, Kang Kang, Yunhui Zhang, and Deming Gou

Figure S1. Construction of hypoxia-induced pulmonary hypertension (HPH) Rat model. – Related to Figure 1

Figure S2. Distribution of various small noncoding RNA (sncRNA) fragment lengths and uniform manifold approximation and projection (UMAP) visualization of distinct sncRNA classes. – Related to Figure 2.

Figure S3. t-distributed stochastic neighbor embedding (t-SNE) analysis of various classes of small noncoding RNA (sncRNA) and a hypoxia-biased miRNA in the brain. – Related to Figure 3

Figure S4. Minimum free energy (MFE) structure of 5 tissue-specific novel miRNA. - Related to Figure 4.

Figure S5. miRNA arm length distribution in normoxia and hypoxia. – Related to Figure 4.

Figure S6. Identification of differentially expressed RNAs (DERNAs) in plasma of hypoxia-induced pulmonary hypertension (HPH) Rats. – Related to Figure 5.

Figure S1. Construction of hypoxia-induced pulmonary hypertension (HPH) Rat model. Body weight (A), right ventricular systolic pressure (RVSP) (B) and right ventricular hypertrophy index (RVHI) (C) of healthy male Sprague-Dawley rats and chronic hypoxia for 21 days. (D) Representative images of hematoxylin and eosin (H&E)-stained lung sections in both HPH and normal rats. (E) Pulmonary arterial wall thickness analysis for HPH and normal rat (n=80 per group).The relative wall thickness was determined as (outer perimeter - inside perimeter)/outer perimeter. NOR, normal control rats; HYP, hypoxia-treated rats. ****indicates p<0.0001; **indicates p<0.01.

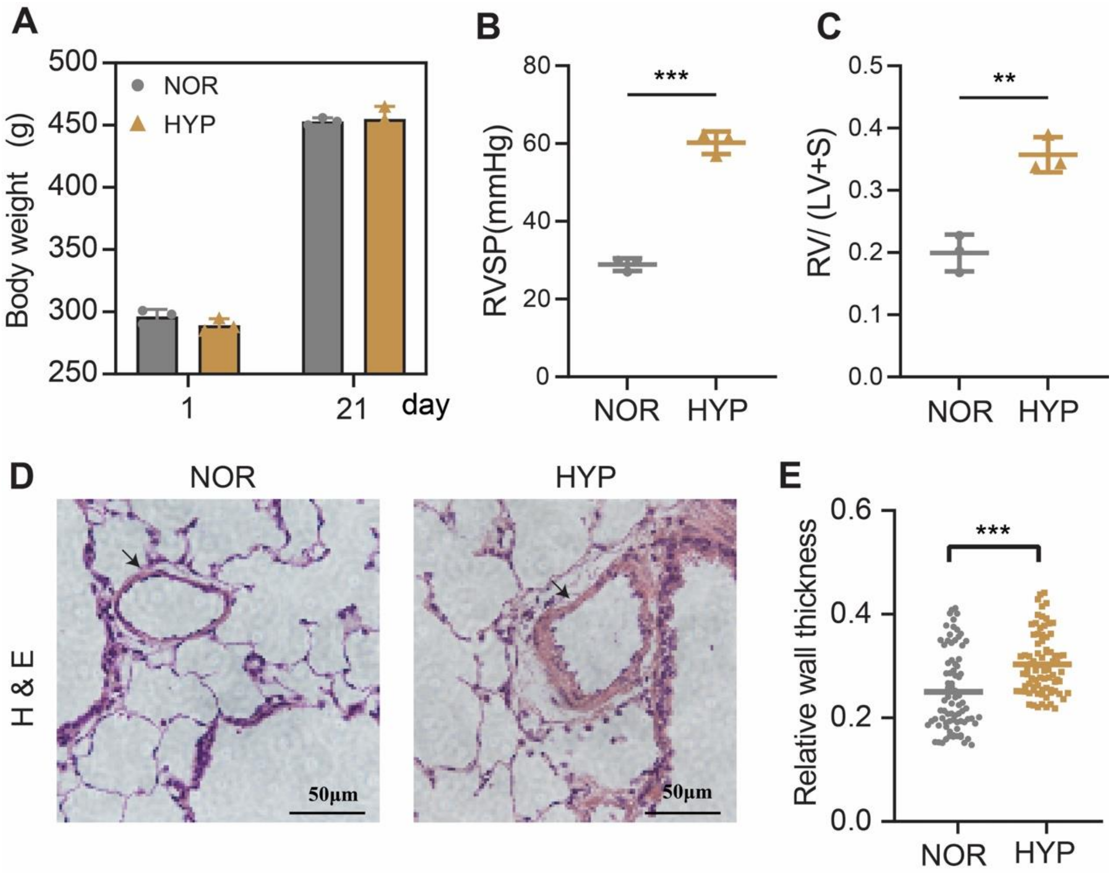

Figure S2. Distribution of various small noncoding RNA (sncRNA) fragment lengths and uniform manifold approximation and projection (UMAP) visualization of distinct sncRNA classes. (A) Fragment length distribution: this panel illustrates the distribution of fragment lengths for various sncRNA classes, including miRNA, piRNA, scaRNA, snoRNA, snRNA, and tDR. (B) UMAP analysis across tissues: UMAP analysis was conducted for different sncRNAs in various tissues, encompassing miRNA, lncRNA, tRNA, Rny, piRNA, and rsRNA in brain, heart, intestine, kidney, liver, lung, spleen, and thymus.

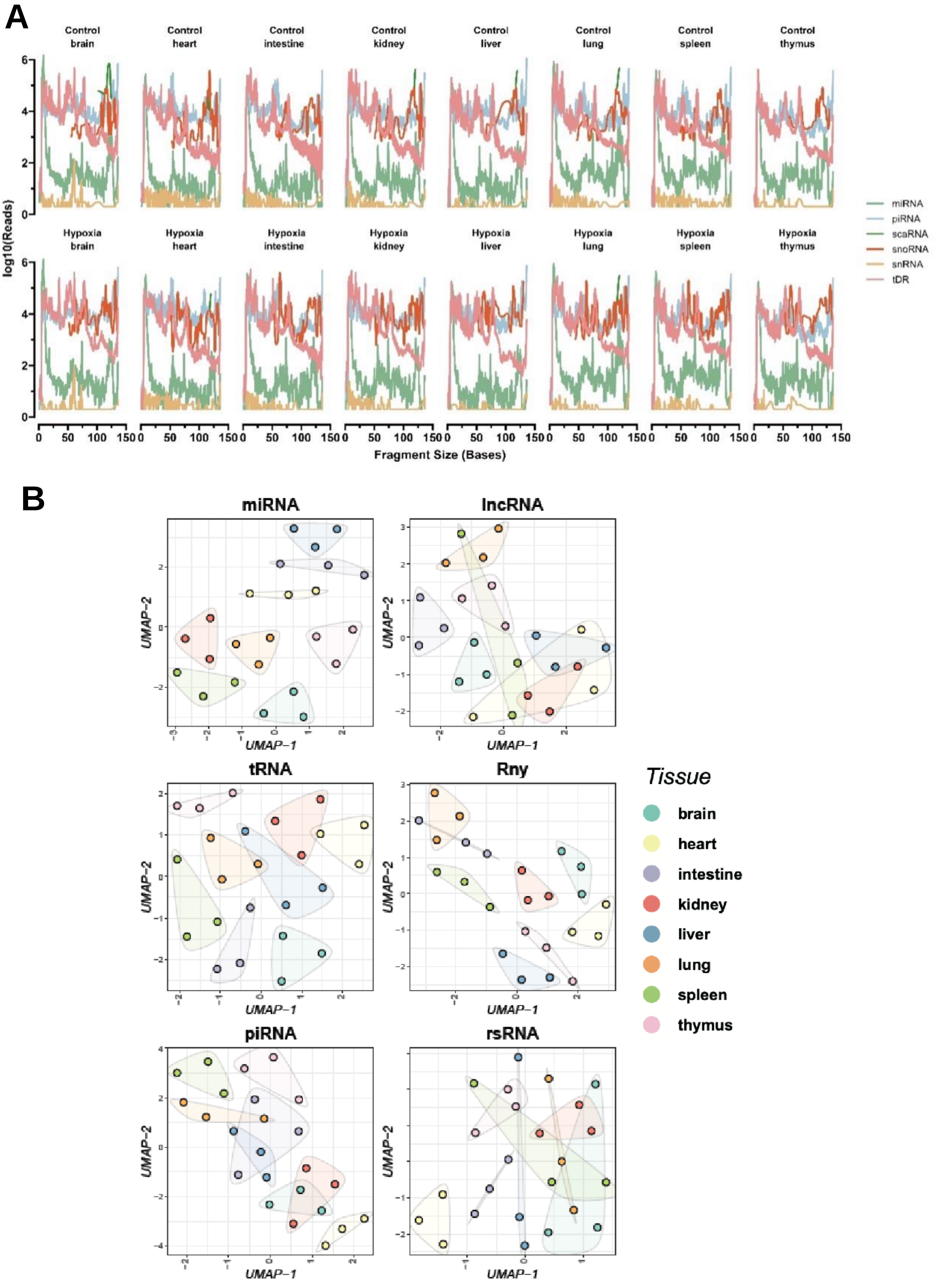

Figure S3. t-distributed stochastic neighbor embedding (t-SNE) analysis of various classes of small noncoding RNA (sncRNA) and a hypoxia-biased miRNA in the brain. t-SNE analysis of all RNA (A), miRNA (B), and tRNA (C). (D) Expression and genomic localization of hypoxia-biased miRNAs. NOR, normal control rats; HYP, hypoxia-treated rats.

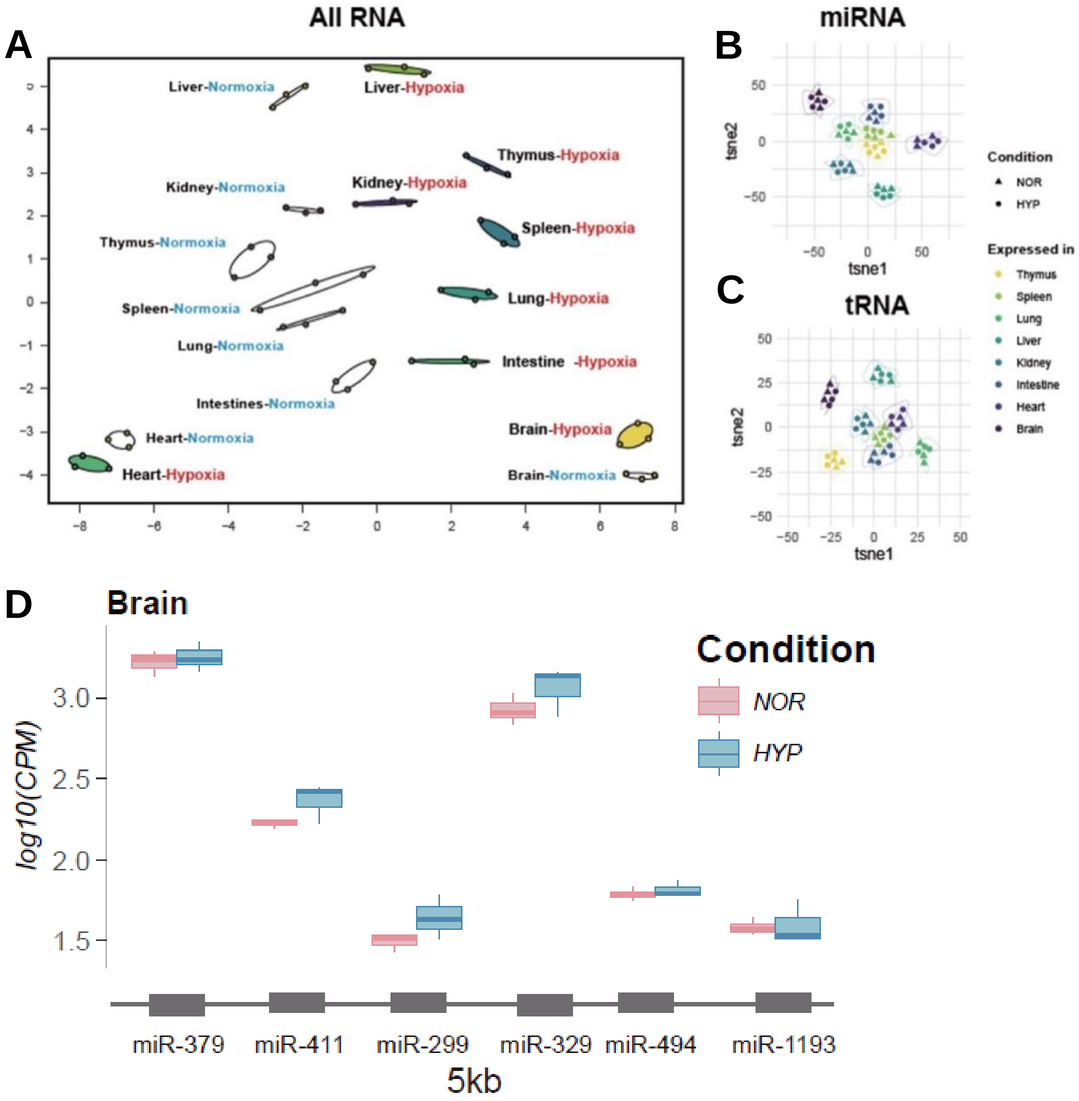

Figure S4. Minimum free energy (MFE) structure of 5 tissue-specific novel miRNA. The structure is colored by base-pairing predicted by RNAFold.

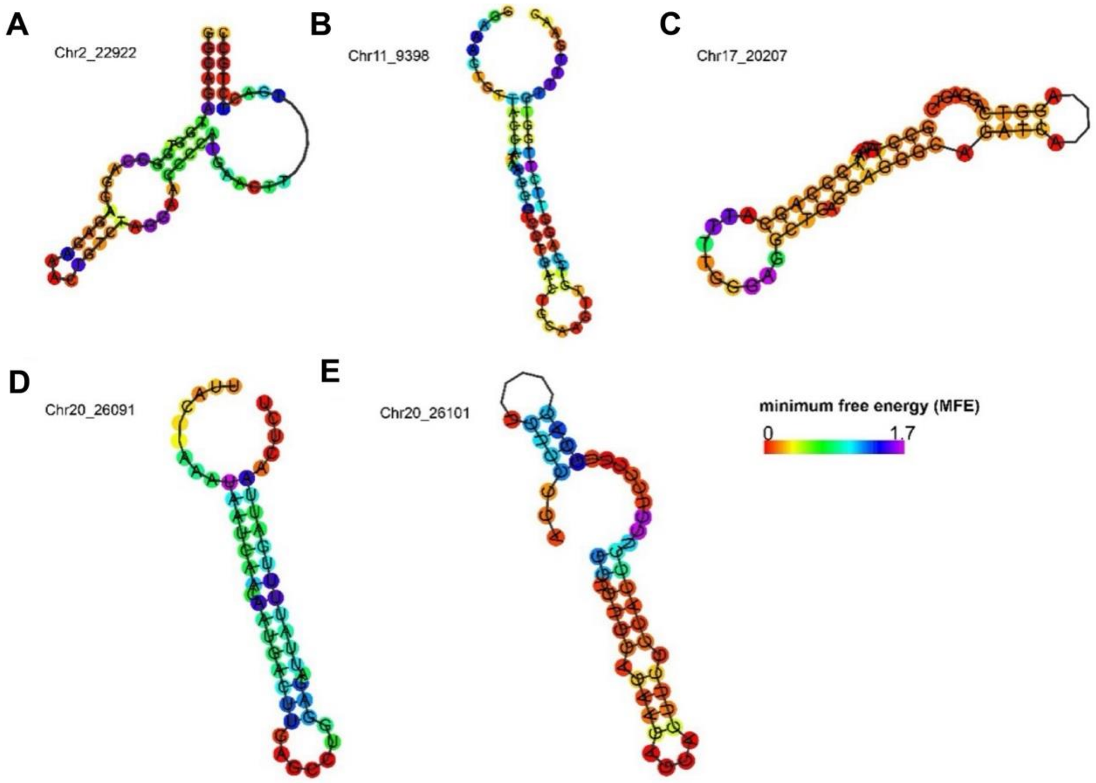

Figure S5. miRNA arm length distribution in normoxia and hypoxia.

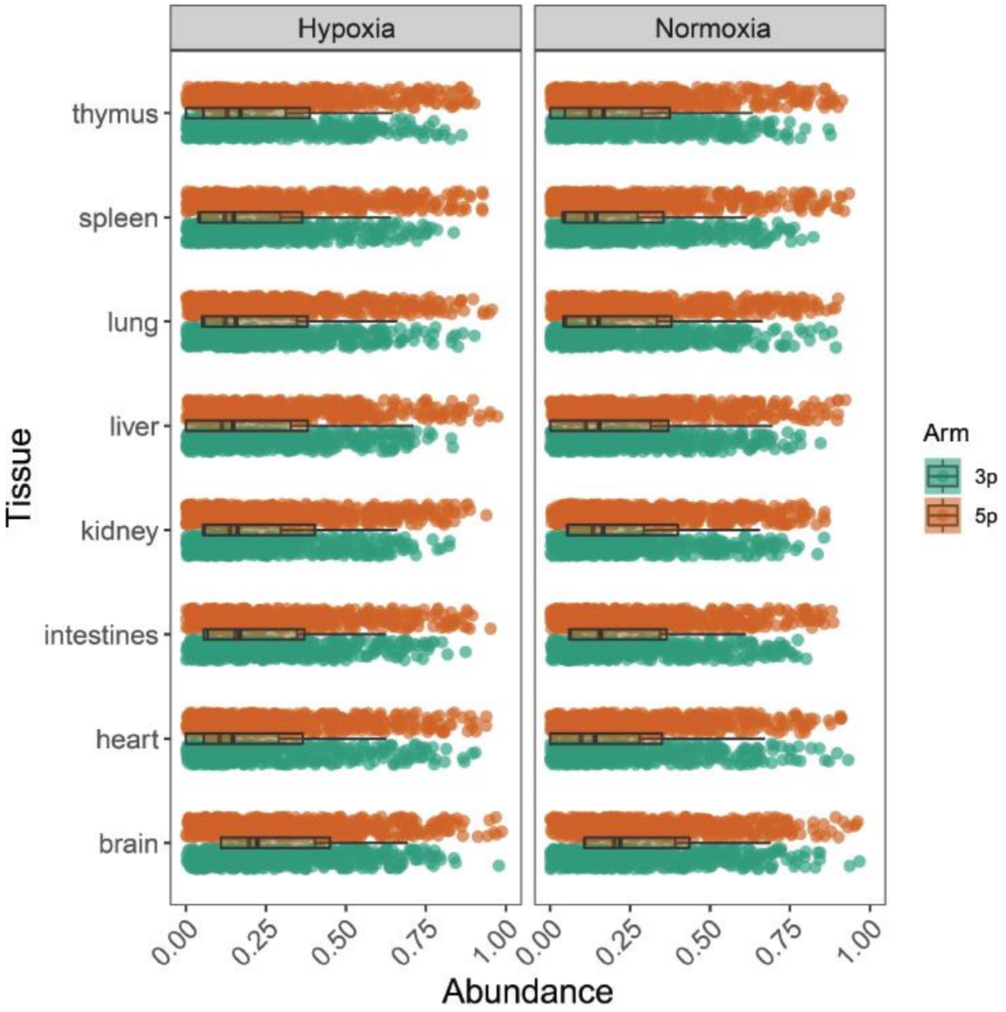

Figure S6. Identification of differentially expressed RNAs (DERNAs) in plasma of hypoxia-induced pulmonary hypertension (HPH) Rats. The volcano plot of DEmiRNAs (A) and DEtDRs (B) between HPH and normal rats. (C) Correlation analysis between the log2Foldchange values of DEmiRNAs identified in plasma and various tissues of HPH rats. **** indicates p < 0.0001; ** indicates p < 0.01; ** indicates p < 0.05.

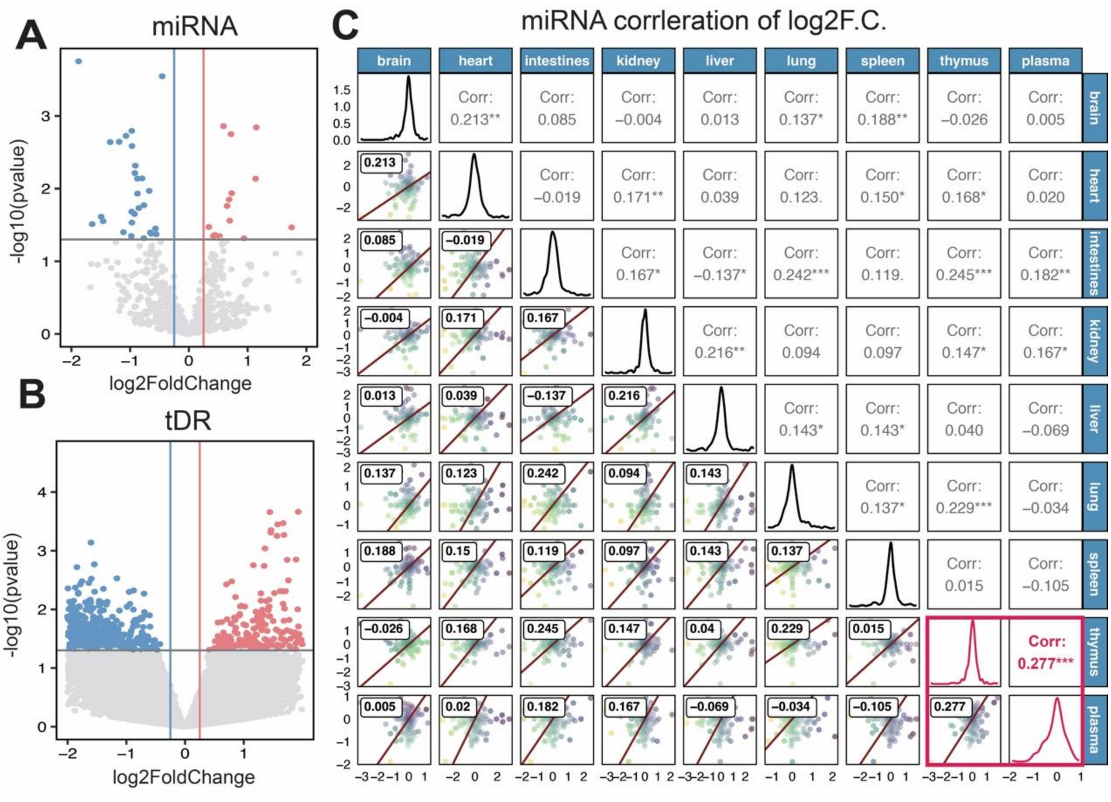

Supplement: Document S1. Figures S1‒S6 [file mmc1.pdf]
